# AMP-Activated Protein Kinase Contributes to Apoptosis Induced by the Bcl-2 Inhibitor Venetoclax in Acute Myeloid Leukemia

**DOI:** 10.3390/cancers13235966

**Published:** 2021-11-27

**Authors:** Noémie Legrand, Amandine Pradier, Laury Poulain, Sarah Mouche, Rudy Birsen, Clément Larrue, Federico Simonetta, Jerome Tamburini

**Affiliations:** 1 Translational Research Centre in Onco-Hematology, Faculty of Medicine, University of Geneva, 1211 Geneva, Switzerland; noemie.legrand@unige.ch (N.L.); amandine.pradier@unige.ch (A.P.); lpoulain@labomgd.ch (L.P.); sarah.mouche@unige.ch (S.M.); rudy_RLR@hotmail.com (R.B.); Clement.Larrue@unige.ch (C.L.); federico.simonetta@unige.ch (F.S.); 2 Geneva University Medical Center, 1205 Geneva, Switzerland; 3 Institut Cochin, Université de Paris, INSERM U1016, F-75014 Paris, France

**Keywords:** AML, AMPK, venetoclax

## Abstract

**Simple Summary:**

The Bcl2 inhibitor venetoclax is a breakthrough therapy in acute myeloid leukemia (AML). We show that venetoclax induces caspase-dependent degradation of AMPK, a central regulator of cellular energy metabolism, with implications in the anti-Leukemic activity of venetoclax in AML.

**Abstract:**

The treatment of acute myeloid leukemia (AML) remains a challenge especially among the elderly. The Bcl-2 inhibitor venetoclax recently showed significant survival benefits in AML patients when combined to low-dose cytarabine or azacitidine. Bcl-2 inhibition initiate mitochondrial apoptosis, but also respiration and cellular ATP production in AML. AMP-Activated Protein Kinase (AMPK) is a central energy sensor activated by increased AMP:ATP ratio to restore the cellular energy balance. Unexpectedly, we observed that venetoclax inhibited AMPK activity through caspase-dependent degradation of AMPK subunits in AML cells. On the other hand, genetic models of AMPK invalidation and re-expression suggested that AMPK participated to the early stages of apoptotic response through a negative regulation of multi-domain anti-apoptotic effectors such as Mcl-1 or Bcl-xL. Together our results suggested a new link between AMPK and Bcl-2-dependent mitochondrial apoptosis that participated to the anti-leukemic activity of venetoclax in AML.

## 1. Introduction

Acute myeloid leukemia results from the proliferation of bone marrow immature myeloid progenitor cells. AML is uniformly treated by intensive chemotherapy in young patients, and with low-dose chemotherapy or hypomethylating agents in older and frail patients [1]. Unfortunately, a vast majority of AML patients especially among the elderly die from this disease [2]. A recent paradigm shift came from the therapeutic use of venetoclax, a highly selective orally available B cell lymphoma 2 (Bcl-2) protein inhibitor [3]. In AML, venetoclax demonstrated on-target activity in preclinical models, and a meaningful efficacy as monotherapy in refractory/relapsed AML patients, and also combined with low-dose cytarabine or azacytidine in previously untreated AML patients [4,5,6,7,8].

Bcl-2 is an anti-apoptotic protein localized to the outer membrane of mitochondria, sequestrating pro-apoptotic effectors such as BAK or BAX and thus preventing the formation of mitochondrial pore for the release of cytochrome c and subsequent apoptosis cascade triggering [9]. In addition, Bcl-2 regulates mitochondrial oxidative metabolism, as venetoclax was shown to inhibit electron transport chain complex I and to decrease oxygen consumption in AML [10]. In AML leukemic stem cells (LSCs) characterized by a low ROS content and a high Bcl-2 expression, venetoclax inhibits oxidative phosphorylation, resulting in the selective eradication of this tumor-promoting cell population, which might contribute to the meaningful activity of venetoclax observed in AML clinical trials [11,12].

While venetoclax inhibits mitochondrial respiration in AML, the consequences of Bcl-2 inhibition on the master intracellular energy sensor AMP activated protein kinase (AMPK) are largely unknown. Heterotrimeric AMPK complexes bind adenosine nucleotides through their γ-subunit, with AMP strongly enhancing AMPK activity, attested by increased AMPKα phosphorylation at Threonine 172 (T172), mediated by liver kinase B1 (LKB1) and calcium/calmoduline dependent protein kinase kinase 1 (CAMKK1) dependent on energy and calcium levels, respectively [13]. Inhibition of ATP production, resulting in an increased AMP:ATP ratio, leads to AMPK activation and to subsequent modulations of metabolic pathways regulating glucose and lipid metabolism, mRNA and protein synthesis, mitochondrial biogenesis and autophagy [13,14]. Overall, AMPK activation favors catabolism, and repress anabolism to generate ATP and maintains cellular energy homeostasis [13].

In AML cells sensitive to apoptosis induction by venetoclax, we observed a drop in intracellular ATP content, but unexpectedly, a rapid decrease of AMPK activity occurred in these cells. In vitro kinase assays excluded that venetoclax directly inhibited AMPK, while instead AMPK degradation was due to the on-target activity of venetoclax through caspase activation in apoptotic cells. Finally, AMPK contributed to the anti-leukemic activity of venetoclax, as AMPK depletion protected venetoclax-treated AML cells from early apoptosis, which correlated with an increased amount of multi-domain anti-apoptotic effectors Mcl-1 and BclxL. Our findings suggested a role for AMPK at the early steps of apoptosis induction by venetoclax in AML.

## 2. Results

### 2.1. Inhibition of AMPK Activity by Venetoclax in AML Cells

**Figure 1:**
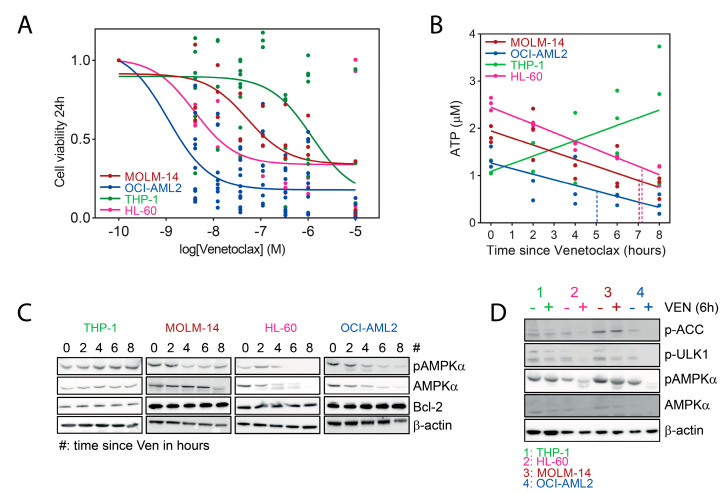
Inhibition of AMPK activity by venetoclax in AML cells. (**A**) Four different AML cell lines were seeded in 384-well plates at 3 × 10^5^ cells/mL and incubated with a dose range of venetoclax during 24 h. Cell viability was then measured using the ATPlite luminescent reagent, and results were analyzed with the nonlinear regression module and plotted using log(inhibitor) versus response (three parameters) function of the Prism software. (**B**) Four different AML cell lines were seeded in 384-well plates at 3 × 10^5^ cells/mL and incubated with 100 nM venetoclax during 0 h o 8 h. Then, ATP content was measured using the ATP lite luminescent reagent. Results of ATP content (Y-axis) were plotted on incubation time (X-axis) and a linear regression was performed using the Prism software. Time for achieving a 50% reduction of ATP content is indicated by vertical dashed lines for each cell lines following their respective color code. (**C**) AML cell lines were seeded at 5 × 10^5^ cells/mL and incubated with 100 nM venetoclax for the indicated times. Western blots were performed using the anti-phospho-AMPKα T172, -AMPKα, -Bcl-2, and -β-actin antibodies. (**D**) AML cell lines were incubated with vehicle (DMSO) or 100 nM venetoclax (VEN) for 6 h, and Western blots were performed using anti-phospho-ACC S79, -phospho-ULK-1 S555, -phospho-AMPKα T172, -AMPKα, and -β-actin antibodies.

Bcl-2 not only constitutively represses the formation of mitochondrial pores and caspase activation, but also participates to mitochondrial respiration and cellular energy homeostasis [11]. We incubated AML cell lines with 100 nM venetoclax during 2 h to 8 h, and selected two highly sensitive (OCI-AML2 and HL-60 with IC50 of 1.1 nM and 4 nM, respectively), one intermediate (MOLM-14, IC50 52.5 nM), and one resistant (THP-1, IC50 1.1 μM) cell lines based on cell viability assays (Figure 1A). Moreover, incubation of AML cells with venetoclax resulted in a decreased intracellular ATP content in sensitive AML cell lines, with a 50% reduction achieved in OCI-AML2, HL-60, and MOLM-14 in 5.2 h, 7 h, and 7 h, respectively, consistent with an inhibition of mitochondrial respiration in these cells (Figure 1B).

As ATP depletion generally favors AMPK activation by increasing the AMP:ATP ratio, we investigated AMPK activity in AML cells incubated with venetoclax during short-term periods. Surprisingly, AMPK phosphorylation at Thr172 was moderately inhibited in MOLM-14, and more strongly in OCI-AML2 and HL-60 cells, respectively, correlating with a proportional decrease of AMPKα amount, while both phospho-AMPK and AMPKα remained unaffected in the THP-1 cell line (Figure 1C). Moreover, AMPK activity, evaluated by the phosphorylation of two direct AMPK substrates acetyl-coA carboxylase (ACC) and Unc-51 like autophagy activating kinase 1 (ULK-1) on Ser79 and Ser555, respectively, was inhibited by a short-term incubation with venetoclax in OCI-AML2 and HL-60 cells, while little to no effect was seen in MOLM-14 and THP-1 cells (Figure 1D, left panel). After 24 h incubation with venetoclax, AMPK activity remained fully inhibited in HL-60 and OCI-AML2 cells, was significantly inhibited in MOLM-14 cells but was unaffected in THP-1 cells (Supplemental Figure S1). Together, these results suggested that short-term incubation with venetoclax inhibited AMPK activity in sensitive AML cells.

### 2.2. Decreased Amount of AMPK Subunits by Venetoclax in AML

**Figure 2:**
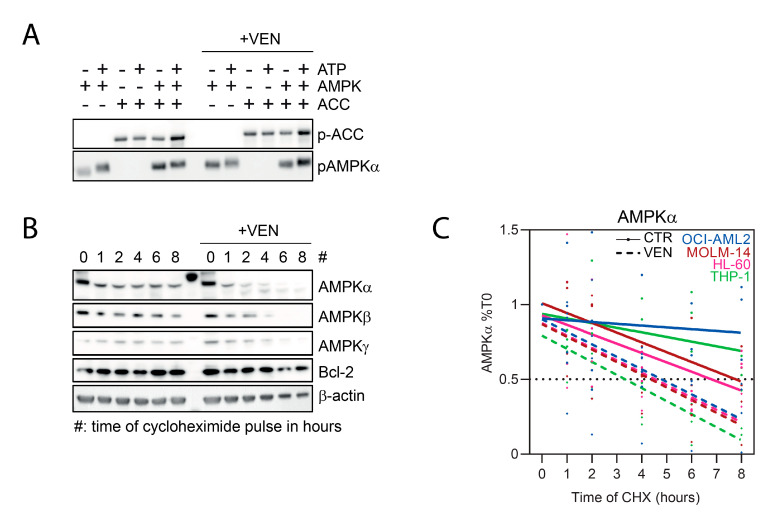
Decreased amount of AMPK subunits by venetoclax in AML. (**A**) Western blots were done from in vitro kinase assay protein mix using anti-phospho-ACC S79 and anti-phospho-AMPK T172 antibodies. (**B**,**C**) AML cell lines were incubated without or with 100 nM venetoclax (VEN), and then submitted to a cycloheximide pulse during the indicated times. (**B**) Western blots were done using anti-AMPKα, β, γ, -Bcl-2, and -β-actin antibodies. (**C**) Quantification of the Western blot signals from three independent experiments using ImaJ software for AMPKα detection in the control (CTR) or venetoclax (VEN) conditions.

We investigated whether venetoclax could directly inhibit AMPK activity in vitro. We first observed that the phosphorylation of recombinant AMPK increased in the presence of ATP, suggesting that AMPK autophosphorylation occurred in vitro (Figure 2A). Moreover, the phosphorylation of recombinant ACC proteins was increased in the presence of AMPK and ATP, showing that direct phosphorylation of ACC by AMPK occurred in vitro (Figure 2A). However, we did not observe a decrease in AMPK autophosphorylation or AMPK-induced ACC phosphorylation, indicating that venetoclax was not a direct AMPK inhibitor (Figure 2A).

As inhibition of AMPK phosphorylation induced by venetoclax correlated to a decreased AMPK protein detection, we performed cycloheximide chase assays [15] to measure the half-life of AMPK α, β, and γ subunits in AML cells incubated with vehicle or venetoclax. Cycloheximide is a protein translation inhibitor, allowing the follow-up of AMPK amounts by immunoblot after Bcl-2 inhibition. While the detection of all three AMPK isoforms was similar across AML cell lines, we readily observed a decreased detection of AMPK α, β, and γ s 4 h of incubation with venetoclax in HL-60 and OCI-AML2 cells, and to a lesser extent in MOLM-14 cells (Supplemental Figure S2A,B). In venetoclax-sensitive AML cell lines (HL-60, OCI-AML2, and MOLM-14), incubation with venetoclax generally decreased the half-life of AMPK α, β, and γ isoforms, with a more marked and constant effect on AMPKα (Figure 2B,C, Table 1 and Supplemental Figure S2A,B). Moreover, the half-life of AMPK subunits was longer in THP-1 cells (not calculable for β and γ subunits), but AMPK degradation was unmasked by venetoclax, suggesting that a minimal activity of venetoclax occurred in these cells resistant to Bcl-2 inhibition (Supplemental Figure S2C,D). Collectively these results suggest that the inhibition of AMPK phosphorylation induced by venetoclax was due to an induction of AMPK protein degradation in AML cells.

### 2.3. AMPK Degradation Is Due to On-Target Caspase Activation by Venetoclax

**Figure 3:**
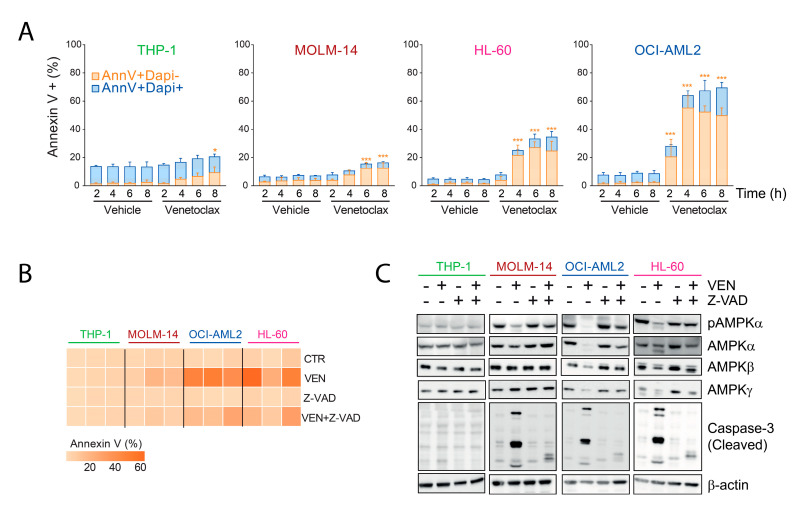
AMPK degradation is due to on-target caspase activation by venetoclax. (**A**) AML cell lines were incubated with 100 nM venetoclax during the indicated times and processed for flow cytometry using annexin V and DAPI staining. Annexin V-positive and DAPI-negative cells are those in early apoptosis, while double positivity indicates either late apoptosis or necrotic cells. The experiment was repeated three times separately. Vertical bars indicate standard deviations. * *p* < 0.05, *** *p* < 0.001. (**B**,**C**) AML cell lines were incubated with vehicle (CTR), 100 nM venetoclax (VEN), 50 µM Z-VAD (pan-caspase inhibitor) or a combination of 50 µM Z-VAD (added 24 h before VEN) and 100 nM venetoclax for 4 h. (**B**) Flow cytometry measurement of annexin V binding done in three separate experiments and plotted in a heat-map format. (**C**) Western blots done using anti-phospho-AMPK T172, -AMPK α, β, γ, -cleaved caspase 3, and –β-actin.

Caspase 3 is the downstream effector of mitochondrial apoptosis activated after Bcl-2 inhibition and subsequent cytochrome c release [9]. From the amino acid sequence of AMPKα1, we detected two potential sites of cleavage by caspase 3, including one after amino acid 518 already described [16] (Supplemental Figure S3A). In OCI-AML2, HL-60, and to a lesser extent in MOLM-14 cell lines, venetoclax induced an increased proportion of annexin V-positive and DAPI-negative cells, corresponding to early apoptosis even after very short-term incubation starting from 2 h (Figure 3A). After 24 h incubation with venetoclax, OCI-AML2 and HL-60 cells were in majority annexin V positive and DAPI positive, in favor of late apoptosis while annexin V+/DAPI- MOLM-14 cells were predominant and still not significantly induced in the THP-1 cell line (Supplemental Figure S3B). Notably, cell cycle was not modified after 24 h incubation with venetoclax in THP-1, MOLM-14, and HL-60, while increased G0/G1 and decreased G1/S proportions were observed in OCI-AML2 cells, probably because of massive apoptosis induction in this highly sensitive cell line (Supplemental Figure S3C). We further observed that pretreatment with the pan-caspase inhibitor Z-VAD [9] significantly reduced annexin V binding in OCI-AML2 and HL-60 cells incubated with venetoclax (Figure 3B). Moreover, Z-VAD prevented the cleavage of caspase 3, and restored the detection of AMPK α, β, and γ subunits in HL-60, OCI-AML2, and MOLM-14 cells treated with venetoclax, while venetoclax did not induce a cleavage of caspase 3 in THP-1 cells (Figure 3C). From these results, we concluded that the inhibition of AMPK phosphorylation observed after incubation of AML cells with venetoclax was due to caspase-dependent degradation of AMPK proteins.

### 2.4. AMPK Contributes to the Pro-Apoptotic Activity of Venetoclax in AML

**Figure 4:**
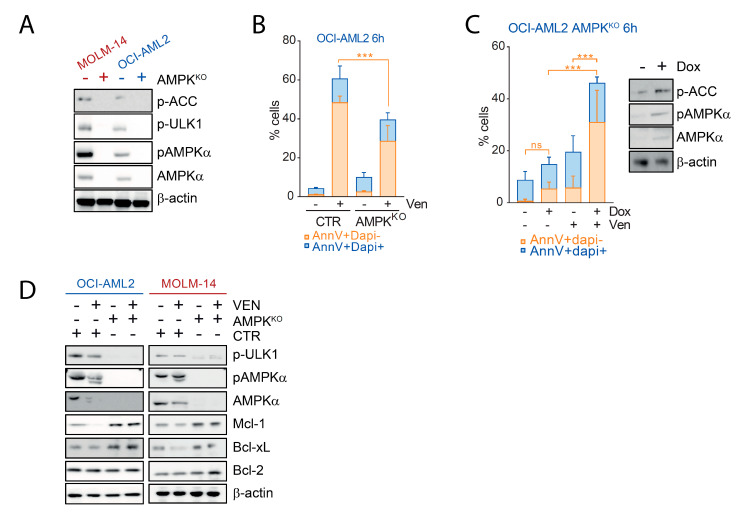
AMPK contributes to the pro-apoptotic activity of venetoclax in AML. (**A**) MOLM-14 and OCI-AML2 cells were transduced with either a control or a *PRKAA1* CRISPR/Cas9 sgRNA targeting AMPKα1 and submitted to Western blots using anti-phospho-ACC S79, -phospho-ULK-1 S555, -phospho-AMPK T172, anti-AMPKα, and anti-β-actin antibodies. (**B**) OCI-AML2 cells expressing (CTR) or not (AMPK^KO^) AMPK were incubated with vehicle or 100 nM venetoclax for 6 h and processed for flow cytometry analysis of apoptosis using annexin V and DAPI staining. (**C**) AMPK^KO^ OCI-AML2 cells were transduced with a vector allowing the expression of AMPKα1 after the addition of doxycycline (Dox). Left panel: cells were incubated with vehicle or 100 nM venetoclax for 6 h and flow cytometry for annexin V and DAPI was performed. Right panel: Western blots were done using anti-phospho-ACC S79, -phospho-AMPK T172, -AMPKα, and –β-actin. (**D**) CTR or AMPK^KO^ MOLM-14 and OCI-AML2 cells were incubated with vehicle or 100 nM venetoclax for 6 h and submitted to Western blotting using anti-phospho-ULK-1 S555, -phospho-AMPK T172, -AMPKα, -Mcl-1, -Bcl-xL, -Bcl-2, and anti-β-actin antibodies. Vertical bars indicate standard deviations. *** *p* < 0.001.

We investigated the role of AMPK in early apoptosis upon Bcl-2 inhibition. We used AMPK deficient MOLM-14 and OCI-AML2 cells generated by CRISPR/Cas9, as reported [17]. We first observed that AMPK^KO^ cells were deficient for AMPK activity as attested by the absence of AMPKα protein expression and of ACC, ULK-1 and AMPKα phosphorylation (Figure 4A). When incubated with venetoclax, OCI-AML2 AMPK^KO^ cells had a lower induction of early apoptosis compared to their CTR counterpart (Annexin V+/DAPI- cells, Figure 4C and Supplemental Figure S4A), and also of late apoptotic events even after 48 h exposure to venetoclax (Annexin V+/DAPI+ cells, Supplemental Figure S4B). A similar trend was observed in MOLM-14 cells, although not reaching statistical significance (Supplemental Figure S4C). Upon AMPK re-expression, AMPK KO cells were significantly targeted by venetoclax, in favor of a positive contribution of AMPK expression in the early apoptotic response induced by Bcl-2 inhibition in AML (Figure 4C). Mechanistically, we observed that short-term incubation with venetoclax decreased Mcl-1 and Bcl-xL protein detection in OCI-AML2 and MOLM-14 cells (Figure 4D). In the same cell lines invalidated for AMPK, Mcl-1 and Bcl-xL detection was increased compared to the control and not modified by venetoclax (Figure 4D). These results suggested that AMPK was required to decrease the amounts of important anti-apoptotic effectors upon Bcl-2 inhibition in AML cells. We next transduced OCI-AML2 AMPK^KO^ cells with a lentiviral vector allowing the conditional expression of AMPKα1 by docycycline (dox). Notably, addition of doxycycline had no impact on the viability of AML cells incubated with venetoclax over a short-term period (data not shown), in contrast to observations made using the same combination during a longer time [10]. These results showed that AMPK expression contributed to early apoptosis induction by venetoclax in AML.

## 3. Discussion

Venetoclax recently emerged as a breakthrough therapy in AML, inducing deep and long-lasting remissions when combined with low-dose cytarabine or hypomethylating agents in patients ineligible for intensive therapy [18]. Understanding the molecular and cellular consequences resulting from Bcl-2 inhibition is essential to design future therapeutic strategies based on venetoclax in AML. In line with previous reports, we observed that venetoclax depleted the intracellular ATP pool in AML cells. As AMPK activity is governed by the intracellular AMP:ATP ratio, we hypothesized that venetoclax could activate AMPK, but surprisingly the opposite was observed. In AML cells sensitive to Bcl-2 inhibition, venetoclax induced a rapid decrease of AMPK phosphorylation and activity, due to a caspase-dependent degradation of AMPK α, β, and γ subunits. To investigate the role of AMPK in the early apoptotic response induced by venetoclax in AML, we depleted AMPK by CRISPR/Cas9 in AML cell lines [17,19].

In contrast to other tissues in which both AMPK α1 and α2 isoforms are expressed, hematopoietic cells lack AMPKα2 expression, even after AMPKα1 invalidation, allowing a complete inhibition of AMPK activity in AML cells with a single CRISPR/Cas9 sgRNA targeting *PRKAA1* [19]. Surprisingly, we observed that AMPK KO cells had a reduced early apoptotic response to Bcl-2 inhibition, which was rescued by AMPK re-expression, showing that AMPK expression contributed to apoptosis induction by venetoclax in AML. Mechanistically, we observed that loss of AMPK was accompanied by an increased Mcl-1 and Bcl-xL detection, which could be explained by an increased translation of these proteins dependent on mTORC1 activation in AMPK KO cells [20,21]. While Mcl-1 and Bcl-xL overexpression was largely involved in resistance to venetoclax [4,22], little is known on the impact of acute Bcl-2 inhibition on the expression of these anti-apoptotic effectors. We observed that short-term incubation of AML cells with venetoclax inhibited Mcl-1 and Bcl-xL protein in control but not in AMPK KO cells, suggesting that this inhibition required AMPK in AML.

While AMPK appeared important at early stages of the apoptotic response induced by venetoclax, increased AMPK activity was detected in tissue samples from chronic lymphocytic leukemia patients relapsing from venetoclax therapy compared to pretreatment samples, suggesting that a deregulation of the metabo-energetic machinery occurred in cells escaping Bcl-2 inhibition [23]. This finding was in agreement with our observation that AMPK complexes were degraded by caspases in AML cells sensitive to Bcl-2 inhibition. We may hypothesize that AMPK degradation prevented the induction of survival pathways such as autophagy in cells primed for apoptosis. Indeed, activated AMPK induces autophagy at least through ULK-1 phosphorylation and mTORC1 inactivation, and also mitophagy through MFF phosphorylation [14]. The role of AMPK in apoptotic response to venetoclax thus appeared dual, dependent on the timing after Bcl-2 inhibition.

AML patients do not uniformly respond to venetoclax, and biomarkers predictive of clinical activity of this agent are actively investigated. Early studies positively and negatively correlated response to venetoclax to Bcl-2 and Bcl-xL or Mcl-1 protein expression, respectively, and to mitochondrial priming by BH3 mimetic peptides ex vivo in tumor cells [4,6]. More recently, AML molecular profile was found to correlate with outcome in patients treated with venetoclax combined to cytarabine or azacytidine, as patients harboring TP53, N/KRAS, SF3B1, or EZH2 mutations had a short survival probability [24]. Moreover, resistance to venetoclax-based combination in patients was associated with an adaptation characterized by an enrichment in clones with activating signaling mutations (RAS or FLT3) or alterations of TP53 [25]. To correlate these genetic markers to the metabo-energetic status of AML cells might represent interesting perspectives to investigate the mechanisms of resistance to venetoclax.

Finally, the role of AMPK in cancer biology remains a matter of active debates. The discovery that *STK11* (encoding LKB1) haploinsufficiency promoted tumors initially suggested that AMPK was a tumor suppressor [26,27]. In contrast, several models revealed that in contrast physiological AMPK activity promoted cancer, such as observed in AML leukemic stem cells characterized by quiescence and a low metabolic activity, in which AMPK invalidation disrupts glucose metabolism and redox balance [28,29]. Particularly, AMPK activity stimulated FIS1-dependent mitophagy that contributed to leukemic stem cell self-renewal, in favor of an oncogenic role of AMPK in this model [29]. Moreover, AMPK inhibition suppressed autophagy induced as a resistance mechanism to bromodomain and extraterminal domain (BET, District of Columbia, WA, USA) inhibitors, and facilitated the anti-leukemic activity of the BET inhibitor JQ1 [30]. We reported that direct pharmacological activation of AMPK was synthetic lethal with mTORC1 activation to induce cytotoxicity in AML cells [19]. Our current finding that AMPK contributed to the early stages of the apoptotic response induced by Bcl-2 inhibition suggested that combining venetoclax to a pharmacological AMPK activator might potentiate the apoptotic response in AML.

## 4. Methods

### 4.1. Cell Lines and Reagents

We used the OCI-AML2, HL-60, THP-1, and MOLM-14 AML cell lines, which were identified by PCR single-locus technology (Promega, PowerPlex21 PCR Kit, Eurofins Genomics, Nantes, France). We also used AMPK knockout (KO) MOLM-14 and OCI-AML2 AML cell lines as previously reported [17,19]. AML cells were cultured in 10% fetal calf serum supplemented minimum essential medium Eagle (MEM, α-modification, Gibco, Life technologies, Carlsbad, CA, USA). We also used HEK293T cells cultured in 10% FBS supplemented Dulbecco’s modified Eagle medium (DMEM, Gibco) with the addition of 250 µg/mL geneticin (G418, Gibco) as lentiviral production packaging cells. Venetoclax was from Selleckchem (Houston, TX, USA), Z-VAD was from Invivogen (San Diego, CA, USA) and cycloheximide was from Sigma-Aldrich (Saint Louis, MO, USA).

### 4.2. AMPK Expression Vector

The synthesis of the human *PRKAA1* gene encoding AMPKα1 was done by GeneArt, and it was cloned in a Gateway pDONR vector (Thermo-Fischer Scientific, Waltham, MA, USA). We then subcloned the *PRKAA1* sequence into the pINDUCER21 doxycycline-inducible vector using the Gateway technology (Thermo-Fischer Scientific, Waltham, MA, USA) (ref pINDCER21). We used lentiviral transduction to express this inducible AMPK expression vector in OCI-AML2 cells after flow cytometry cell sorting on GFP.

### 4.3. Cell Viability Assay

Cells were seeded in 40 μL per well in 384-well plates at the density of 0.3 × 10^6^ cells/mL for 48 h with vehicle (the highest concentration of DMSO), or different concentrations of venetoclax (log[venetoclax]: −8.38, −7.91, −7.43, −6.95,−6.47, −6, and −5). A robot distributed 40 μL of the ATPlite luminescence assay reagent (ATPlite Luminescence Assay System; Perkin Elmer, Walthman, MA, USA) in each well, and luminescence was recorded using a SpectraMax L384LW reader (Molecular Devices, San Jose, CA, USA). Luminescence data in rectified linear units (RLU) were processed with background noise removal (ATPlite signal in the absence of cells) and outlier elimination using the modified Thompson Tau test. Data were then normalized to the vehicle-treated conditions.

### 4.4. ATP Quantification

Cells were incubated with vehicle (DMSO) or 100 nM of venetoclax in 384 wheel plates during 0 h to 8 h. At the end of each incubation, 20 μL of ATPlite luminescence assay reagent was distributed in each well for a final volume of 40 μL, which were homogenized for 2 min at 1100 rpm on an orbital shaker (OrbiShaker MP, Benchmark Scientific, Sayreville, NJ, USA) and incubated for 5 min at room temperature to stabilize luminescent signals. Luminescence was recorded using a SpectraMax L384LW reader with an acquisition time of 0.2 s. The units of luminescent signal were proportional to the intracellular ATP content and were correlated to the luminescent signal obtained from serial dilution of ATP done on the same plates.

### 4.5. Western Blots

Cells were lysed in 100 μL 1X Laemmli buffer [62.5 mM Tris HCl pH 6.7, 10% glycerol, 2% sodium dodecylsulfate (SDS), 24 mM dithiotreitol (DTT), 2 mM Vanadate, bromophenol blue], heated at 90 °C for 5 min, and resolved by SDS-polyacrylamide gels electrophoresis, transferred to nitrocellulose membranes, and probed with primary antibodies. Protein signals were revealed by chemoluminescence (SuperSignal^TM^ West Pico PLUS, Thermo-Fisher Scientifics) and detected using a CCD camera (Fujifilm LAS-3000 Imager, Fuji, Tokyo, Japan). Quantification of Western blots signal intensities was done using the ImageJ software. Antibodies used are listed below (Table 2).

### 4.6. In Vitro Kinase Assays

We incubated 200 ng of human recombinant AMPK and ACC proteins (Abcam in a kinase buffer (Cell Signaling Technologies, Danvers, MA, USA) at 37 °C for 30 min without or with 100 µM ATP (Sigma-Aldrich, St. Louis, MO, USA). Enzymatic reactions were then stopped by 20 mM EDTA, then solubilized in Leammli sample buffer (1X), and submitted to immunoblotting.

### 4.7. Flow Cytometry

Cells were washed in annexin V binding buffer, incubated with Phycoerythrin-coupled annexin V (BD biosciences) and 1/100 4’, 6-diamidino-2-phénylindole (DAPI), as reported. Flow cytometry assays were analyzed using the Flowjo software (BD biosciences, Franklin Lakes, NJ, USA).

### 4.8. Statistics

The number of independent replicates of each experiment is indicated in the figures legends as *n* = value. All experiments were repeated at least three time separately. Differences between the mean values of two experimental groups were analyzed using the two-tailed Student’s t test or a Mann-Whitney test in case of nonparametric data. In comparisons involving more than two groups, we used analysis of variance (ANOVA) and Tukey’s multiple comparison tests. Statistical analyses were performed using Prism software 8.1.1 (GraphPad, San Diego, CA, USA).

## 5. Conclusions

We showed that Bcl2 inhibition by venetoclax induced caspase-dependent AMPK degradation in AML. Moreover, AMPK could participate to early apoptosis induced by venetoclax through the regulation of apoptotic effectors in AML. These results add a new layer in our understanding of the therapeutic activity of venetoclax in AML.

## Figures and Tables

**Table 1:** Quantification of AMPK subunits half-life. Cells were submitted to a cycloheximide chase, and then AMPK subunits amount was assessed by Western blot and quantified using ImaJ. Protein half-life was calculated using the regression function of AMPK subunit detection dependent on time. N/E: not evaluable.

Cell Lines	AMPKα	AMPKβ	AMPKγ
OCI-AML2	34 h	18.9 h	16.2 h
OCI-AML2 VEN	4.8 h	4.8 h	4 h
MOLM-14	7.7 h	40.7 h	9 h
MOLM-14 VEN	4.35 h	5.5 h	9 h
HL-60	4.8 h	10.5 h	11.6 h
HL-60 VEN	4.5 h	7.5 h	7.8 h
THP-1	14 h	N/E	N/E
THP-1 VEN	3.3 h	N/E	8 h

**Table 2:** Antibodies used for Western blot.

Antibody	Manufacturer	Reference
β-actin	Sigma-Aldrich	A-74
AMPKα	Cell signaling	2532
AMPKα1	Cell signaling	2795
AMPKβ	Cell signaling	12,063
AMPKγ	Cell signaling	4187
Phospho-ACC (S79)	Cell signaling	3661
Phospho-ULK-1 (S555)	Cell signaling	5869
Phospho-AMPK (T172)	Cell signaling	4188
Bcl-2	Cell signaling	4223
Bcl-xL	Cell signaling	2762
Mcl-1	Cell signaling	94,296
Caspase 3	Cell signaling	9662
PARP	Cell signaling	9542
rabbit HRP-linked IgG	Cell signaling	7074
mouse HRP-linked IgG	Cell signaling	7076

## Data Availability

The data presented in this study are available in this article and supplementary material.

## Supplementary Materials

The following are available online at https://www.mdpi.com/article/10.3390/cancers13235966/s1, Figure S1. Inhibition of AMPK activity by venetoclax in AML cells; Figure S2. Decreased expression of AMPK subunits by venetoclax in AML; Figure S3. AMPK degradation is due to on-target caspase activation by venetoclax; Figure S4. AMPK contributes to the pro-apoptotic activity of venetoclax in AML; Figure S5: Original blots.
