# Supplementary material for: AMP-Activated Protein Kinase Contributes to Apoptosis Induced by the Bcl-2 Inhibitor Venetoclax in Acute Myeloid Leukemia
Source: Cancers (Basel). 2021 Nov 27;13(23):5966. doi: 10.3390/cancers13235966 (PMC8656606; doi:10.3390/cancers13235966)
Supplement: Supplementary file 1 [file cancers-13-05966-s001.zip › cancers-1400578-supplementary.pdf]

cancers

Supplementary Files

AMP-Activated Protein Kinase Contributes to Apoptosis Induced by the Bcl-2 Inhibitor Venetoclax in Acute Myeloid Leukemia

Noemie Legrand, Amandine Pradier, Laury Poulain, Sarah Mouche, Rudy Birsen, Clement Larrue, Federico Simonetta and Jerome Tamburini

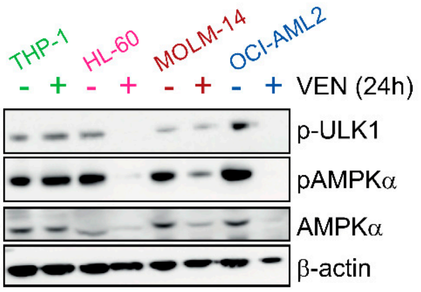

Figure S1. Inhibition of AMPK activity by venetoclax in AML cells. A. Four AML cell lines were incubated without or with 100nM venetoclax for 24h and Western blots were performed with anti-phospho-ULK-1 S555, -phospho-AMPKα T172, -AMPKα and –β-actin antibodies.

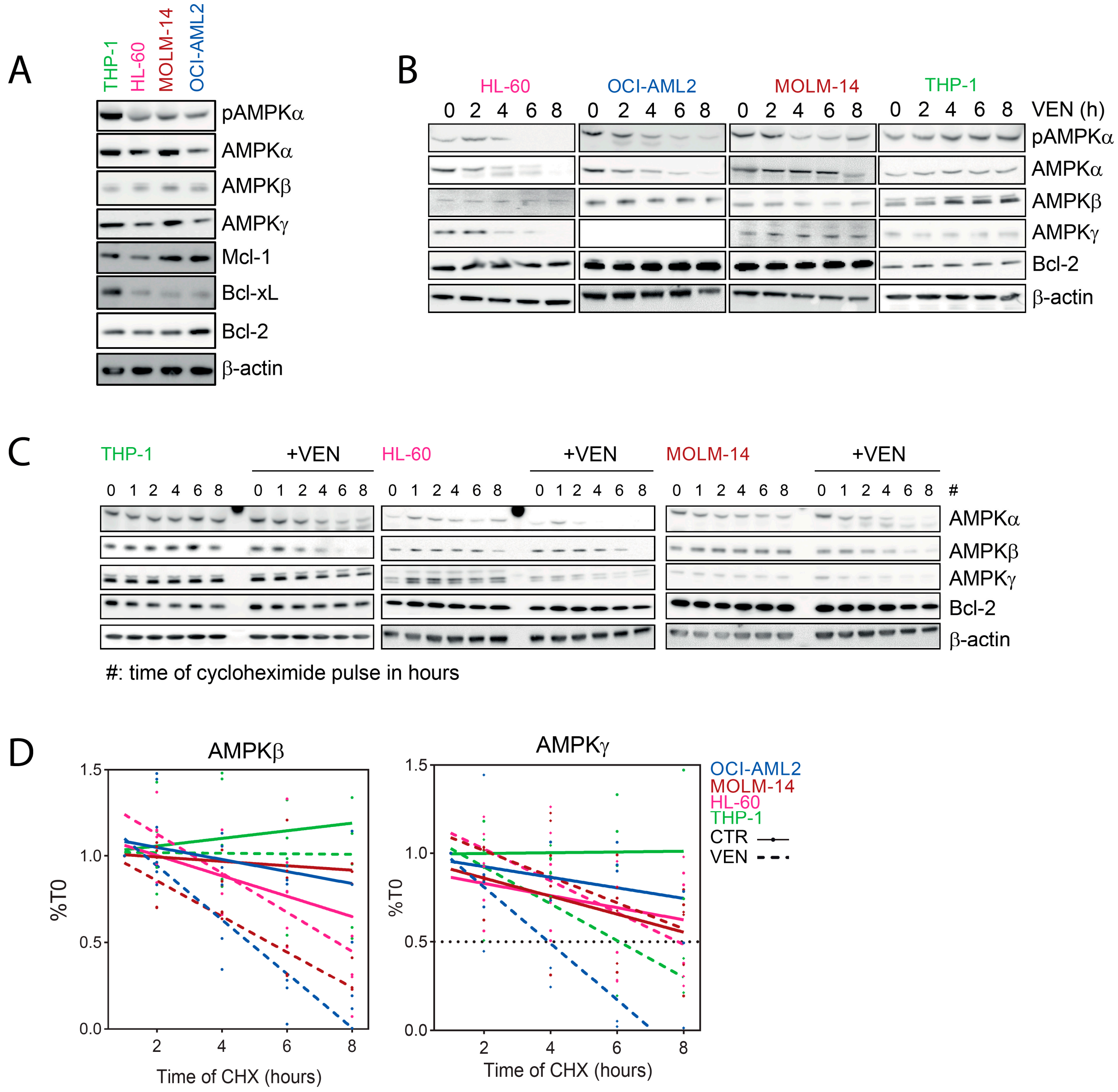

Figure S2. Decreased expression of AMPK subunits by venetoclax in AML. A. Western blots were done in four AML cell lines using anti-phospho-AMPK T172 , -AMPKα, β, γ, -Mcl-1, -Bcl-xL, -Bcl-2 and -β-actin antibodies. **B.** AML cell lines were incubated without or with 100nM venetoclax (VEN)for the indicated times, and Western blots were done using anti-phospho-AMPK T172, -AMPKα, β, γ, -Bcl-2 and -β-actin antibodies. **C-D.** AML cell lines were incubated without or with 100nM venetoclax (VEN), and then submitted to a cycloheximide pulse using 10µg/ml cycloheximide during the indicated times. **C.** Western blots were done using anti-AMPKα, β, γ, -Bcl-2 and -β-actin antibodies. **D.** Quantification of the Western blot signals from three independent experiments using ImaJ software for AMPKα expression in the control (CTR) or venetoclax (VEN) conditions.

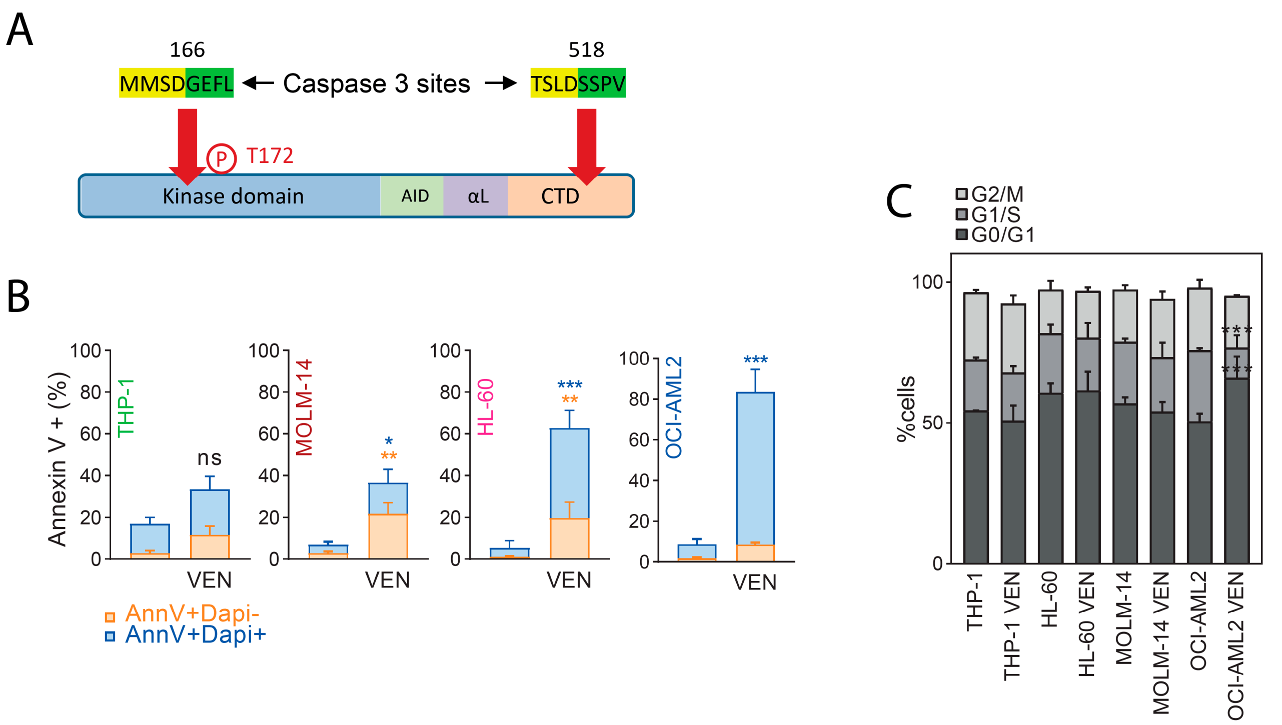

Figure S3. AMPK degradation is due to on-target caspase activation by venetoclax. A. Schematic representation of AMPKα subunit including the kinase domain, the auto-inhibitory domain (AID), the α-linker (α-L) domain and the c-terminal domain (CTD), with the two caspase 3 cleavage sites at residues 166 and 518 figurated. **B.** AML cell lines were incubated without or with 100nM venetoclax (VEN) for 24h and annexin V and DAPI staining was done by flow cytometry. **C.** AML cell lines were incubated without or with 100nM venetoclax (VEN) for 24h and cell cycle was evaluated by flow cytometry propidium iodine staining. Vertical bars indicate standard deviations. *p<0.05, **p<0.01. ***p<0.001.

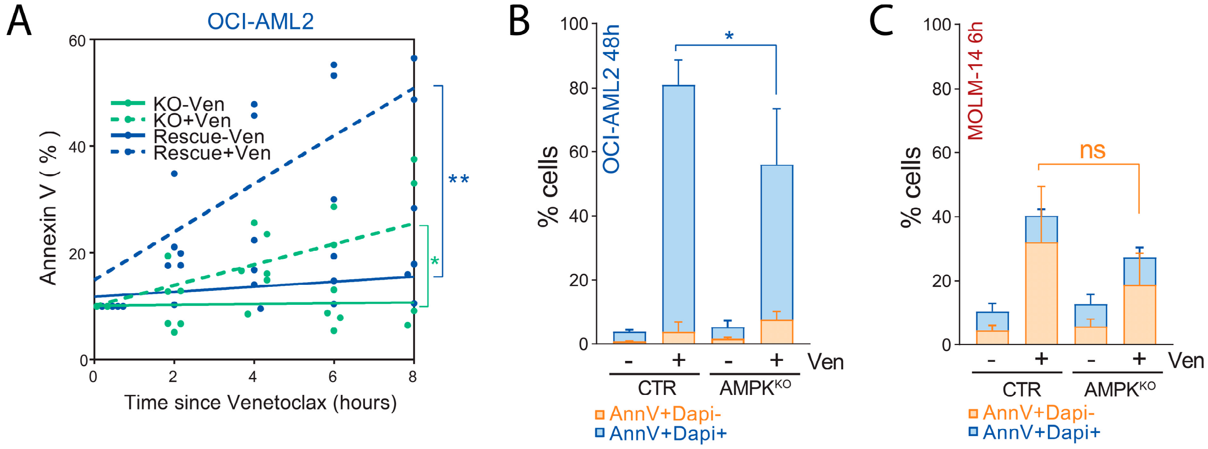

Figure S4. AMPK contributes to the pro-apoptotic activity of venetoclax in AML. A. AMPK^{KO} OCI-AML2 cells were transduced with a vector allowing the expression of AMPKα1 after the addition of doxycycline (Dox), referred to as rescue. Cells were incubated with vehicle or 100nM venetoclax for the indicated times and flow cytometry for annexin V was performed. Results are presented as a linear regression of annexin V positivity dependent on time. **B.** OCI-AML2 CTR or AMPK^{KO} cells were incubated without or with xxnM venetoclax for 48h and annexin V and DAPI staining were measured by flow cytometry. **C.** OCI-AML2 CTR or AMPK^{KO} cells were incubated without or with 100nM venetoclax for 6h and annexin V and DAPI staining were measured by flow cytometry. Vertical bars indicate standard deviations. *p<0.05, **p<0.01. ***p<0.001, ns: not significant.

Related to Figure 1C

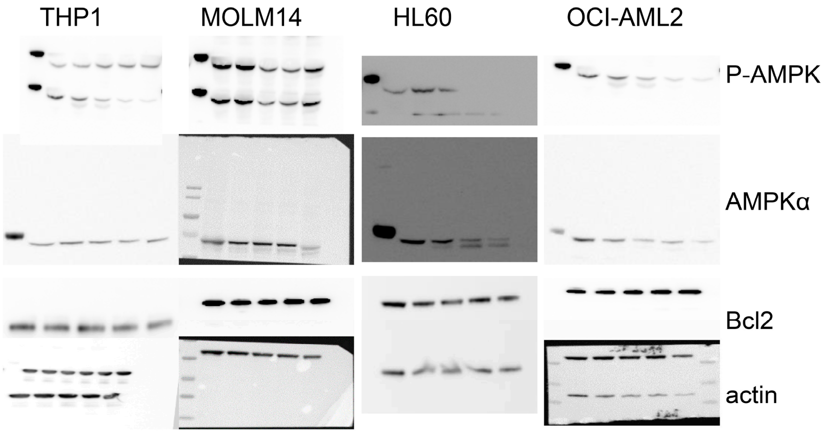

Related to Figure 1D

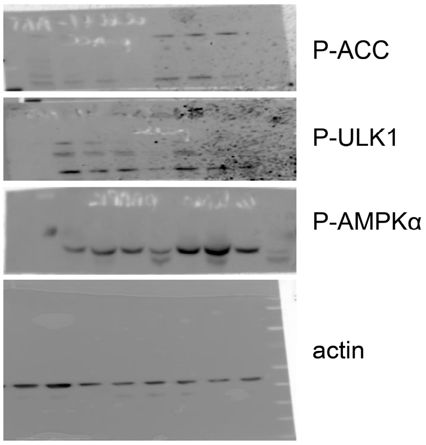

Related to Figure 2A

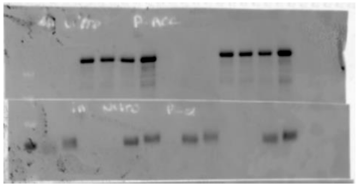

Related to Figure 2B

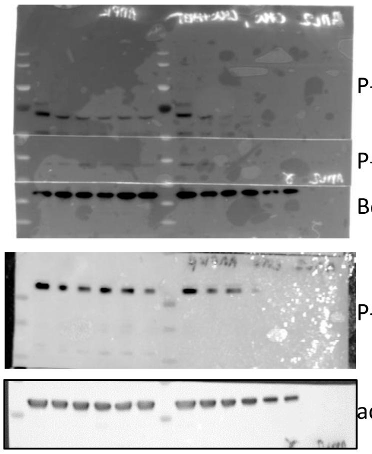

Related to Figure 3C

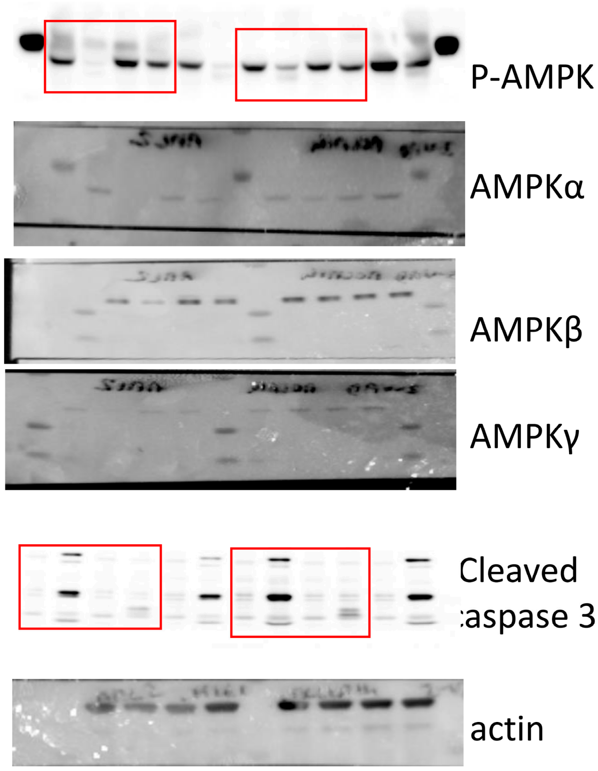

Related to Figure 3C

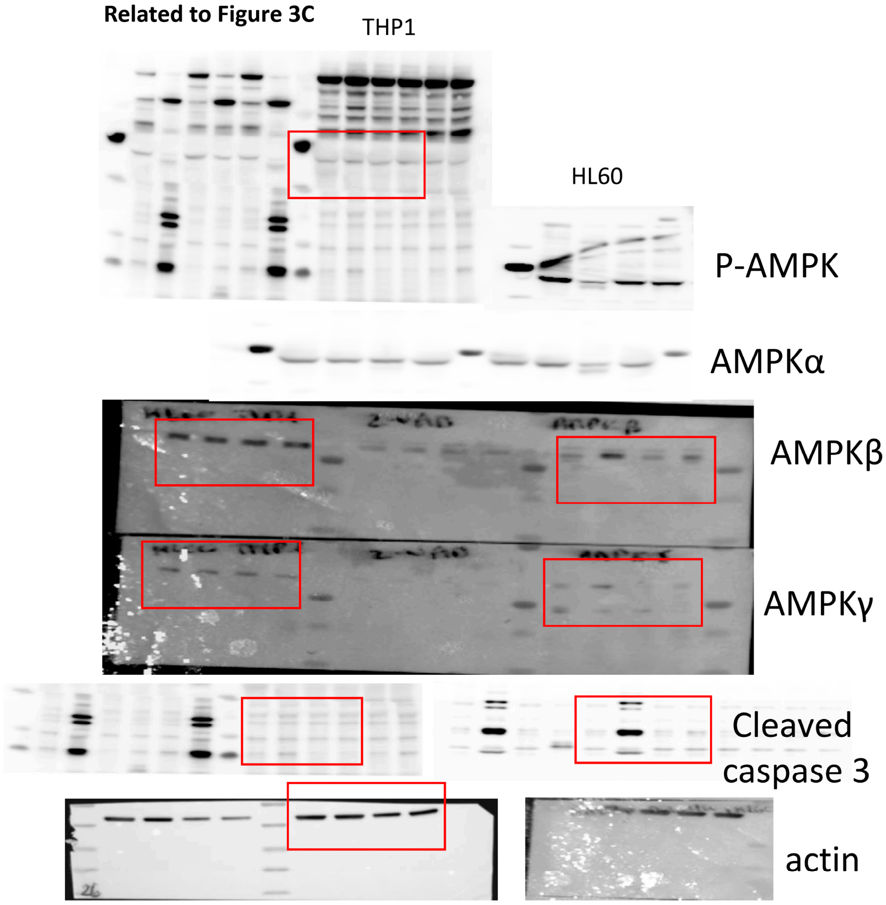

Related to Figure 4A

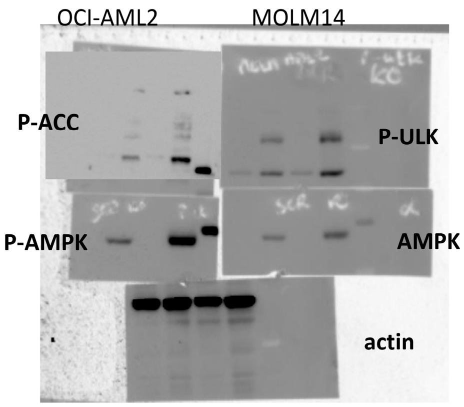

Related to Figure 4B

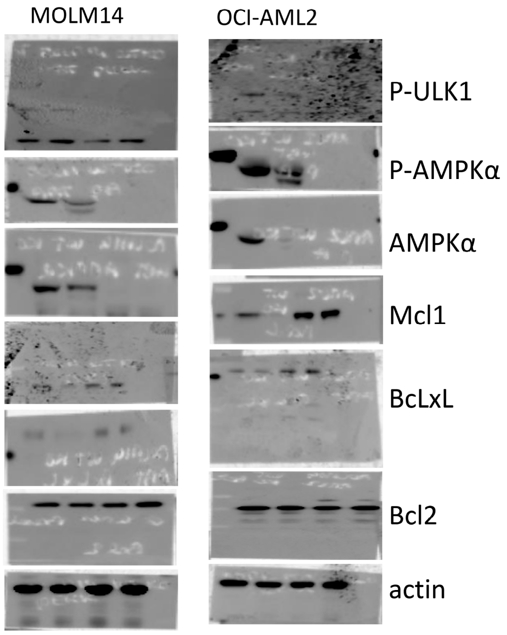

Related to Figure 4C

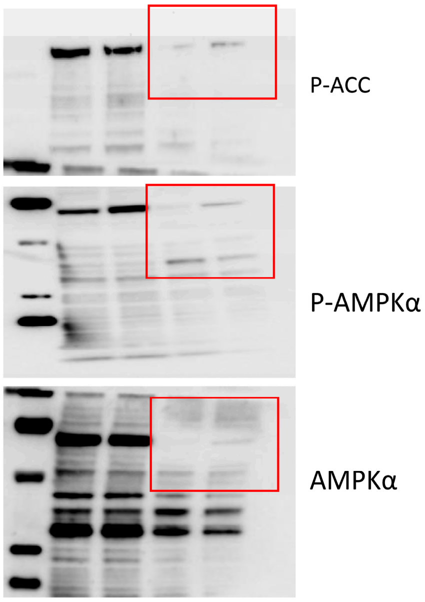

Figure S5. Original Western blots.
